# Supplementary material for: Automatic mechanistic inference from large families of Boolean models generated by Monte Carlo tree search
Source: Front Cell Dev Biol. 2023 Aug 25;11:1198359. doi: 10.3389/fcell.2023.1198359 (PMC10485623; doi:10.3389/fcell.2023.1198359)
Supplement: Supplementary file 1 [file DataSheet1.PDF]

Supplementary Methods

MCTS Implementation

MCTS has four phases: selection, expansion, rollout, and backpropagation. During selection, MCTS recursively descends the search tree, choosing the child branch with the highest upper confidence bound (UCT), the calculation of which is described below. Upon reaching a leaf node, MCTS expands the leaf by creating a new child model for all possible interactions that can be added to the model corresponding to the leaf of the search tree.

These possible interactions are pulled from a list we call the action list. After expanding the rule set of a child node with a new interaction, we remove all conflicting inhibiting and activating interactions from the interaction list of the child. This prevents a source species from both activating and inhibiting a single target species in the model. Users can apply more complex manipulations to the list of possible interactions to inject their prior knowledge of the system. For example, one could restrict possible interactions to only those in a database of transcription factor and gene pairs or from results of a tandem affinity purification experiment. Further, one could limit the number of interactions that a species participates in by removing all interactions from the action list that include the given species once a limit is reached. Each possible interaction has a prior probability of being selected. These priors could be manually specified to bias the search towards more biologically plausible interactions. The priors are multiplied against the random probabilities that are sampled during selection and random rollouts.

After selecting and then expanding a leaf node, MCTS performs a random rollout. This consists of randomly adding interactions to the model, stopping when a special "stop" action is chosen from the interaction list. The "stop" action has a manually chosen prior probability that can be varied to lengthen or shorten random rollouts. After stopping, the resulting model is simulated and scored for similarity. The purpose of the random rollout is to provide a stochastic estimate of the best similarity that can be achieved by models expanded from a given branch of the search tree.

This estimate is improved through the backpropagation process. Each node in the search tree maintains two statistics: number of visits (N_v) and best similarity (D^*). N_v is the number of times the selection step has chosen a branch containing the given node. After simulating and scoring a rollout, MCTS ascends the search tree from the leaf towards the root. At each node in this path, we increment the number of visits, N_v. If the similarity of the rollout $\mathcal{D}$ is greater than D^* then we set $D^* \coloneqq \mathcal{D}$. These statistics are used to calculate the upper confidence bound, which guides the selection process.
$$

\text{UCT} = \mathcal{D}^{*} + c\sqrt{\frac{\ln{N_v(n_i)}}{N_v(n_j)}}
$$
where $N(n_i)$ is the number of visits of the current node n_i and its child node n_j. c is an exploration constant. The exploration constant is a hyperparameter that balances exploration vs exploitation in the search. We use an exploration constant that decays as the search progresses, favoring early diversity in the search but exploitation of good branches at later iterations.

One cycle of selection, expansion, rollout and backpropagation constitutes an iteration of the algorithm. The user can choose a fixed number of iterations or a time limit at which to halt the search. Once this limit is reached, the selection procedure is run from the root node of the search tree, effectively choosing the branch that yielded the best models. We then restart the search after adding the selected interaction to a base model. By frequently taking a step and restarting the search with a model that already contains good interactions, we allow the search to efficiently probe deeper into the search space, following branches that yield good results.

At each rollout we persist the generated model to storage along with its similarity score. Thus, the MCTS algorithm is "anytime", i.e. it returns valid (but not necessarily perfect) models immediately. We parallelize the search by simply running the search on parallel processes with no communication. This means that each process constructs a search tree independently. This prevents information sharing between search processes and introduces potential redundancy in the individual searches and simulations. This loss of performance is balanced by the reduction of communication and synchronization overhead in maintaining consistency in a shared search tree, while also allowing for greater diversity in the individual search processes.

MCTS Enhancements

Rapid Action Value Estimation (RAVE)

In standard MCTS, the estimated best value of a given node (i.e. adding a specific interaction to the Boolean model) is computed by backpropagating similarity scores from all random rollouts below that node in the search tree. Thus, the node's value estimate is limited to rollouts from its branch of the tree. However, other branches of the search tree may include the same action. Rollouts from the other branches could provide some amount of information about the value of the action on other branches. This is the motivating insight of RAVE. RAVE maintains a list of actions with their number of visits and best values, accumulated across all branches. During selection, a node's value is calculated as a weighted average of the RAVE value and the standard (branch specific) value.

$$\hat{\mathcal{D}} = (1 - \beta(n_i, k))\mathcal{D}^{*} + \beta(n_i, k)\mathcal{D}^{rave}$$

Here, D^* is the branch-specific value, D^{rave} is the accumulated RAVE value, and $\hat{D}$ is the weighted average. The weighting factor β is a heuristically determined function of the number of visits to search tree node n_i and a parameter k that determines the number of visits when D^* and D^{rave} are weighted equally:

$$\beta(n_i, k) = \sqrt{\frac{k}{3N_v(n_i) + k}}$$

The weighted average is then substituted into the UCB calculation during selection:

$$\mathrm{UCB} = \hat{\mathcal{D}} + c\sqrt{\frac{\ln N_v(n_i)}{N_v(n_j)}}$$

Nested Search

In standard MCTS, after reaching the iteration limit, we add the best interaction to our model and restart the search process. As a result, information from the previous search steps is lost. We remedy this by retaining the sequence of actions taken by the rollout with the highest reward. If any subsequent search steps fail to find a rollout with higher similarity, then we take the next action from the previous best rollout sequence. This technique is known as nested MCTS.

Branch Retention

As explained above, nested search allows MCTS to retain information from previous search steps about the best sequence of actions. However, the statistics stored at each node of the tree are lost at each step. We can retain the search statistics for the branch chosen at each step, an option we refer to as "branch retention".

Supplementary Table S1 - Number of attractors in randomly sampled models

	Stable		Cyclic		
	Mean±Std	Median	Mean±Std	Median	Cycle length
8 species	2.6±1.11	3.00	2.90±1.75	3.00	2.65±0.91
16 species	2.4±1.30	2.00	3.59±2.47	3.00	3.37±1.91
32 species	2.4±1.39	2.00	4.42±2.72	4.00	5.36±6.83

Supplementary Table S2 - Active/Inactive ratio of attractors from sampled models

	Stable		Cyclic	
	Mean±Std	Median	Mean±Std	Median
8 species	0.3±0.27	0.33	0.33±0.20	0.30
16 species	0.2±0.21	0.19	0.28±0.17	0.30
32 species	0.2±0.20	0.15	0.30±0.15	0.30

Supplementary Table S3 - Segment Polarity Network Reference Rules

$$SLP_i^{t+1} := \begin{cases} 0 & \text{if i} \in \{0,2\} \\ 1 & \text{if i} \in \{1,3\} \end{cases}$$

$$\begin{aligned}
wg_i^{t+1} &:= (\mathrm{CIA}_i^t \text{ and } \mathrm{SLP}_i^t \text{ and not } \mathrm{CIR}_i^t) \text{ or } [\mathrm{wg}_i^t \text{ and } (\mathrm{CIA}_i^t \text{ or } \mathrm{SLP}_i^t) \text{ and not } \mathrm{CIR}_i^t] \\
&= (\mathrm{CIA}_i^t \text{ and } \mathrm{SLP}_i^t \text{ or } \mathrm{wg}_i^t \text{ and } \mathrm{CIA}_i^t \text{ or } \mathrm{wg}_i^t \text{ and } \mathrm{SLP}_i^t) \text{ and not } \mathrm{CIR}_i^t \\
WG_i^{t+1} &:= \mathrm{wg}_i^t \\
en_i^{t+1} &:= (\mathrm{WG}_{i-1}^t \text{ or } \mathrm{WG}_{i+1}^t) \text{ and not } \mathrm{SLP}_i^t \\
EN_i^{t+1} &:= \mathrm{en}_i^t \\
hh_i^{t+1} &:= \mathrm{EN}_i^t \text{ and not } \mathrm{CIR}_i^t \\
HH_i^{t+1} &:= \mathrm{hh}_i^t \\
ptc_i^{t+1} &:= \mathrm{CIA}_i^{t+1} \text{ and not } \mathrm{EN}_i^t \text{ and not } \mathrm{CIR}_i^t \\
&= \mathrm{CIA}_i^{t+1} \text{ and not } (\mathrm{EN}_i^t \text{ or } \mathrm{CIR}_i^t) \\
PTC_i^{t+1} &:= \mathrm{ptc}_i^t \text{ or } (\mathrm{PTC}_i^t \text{ and not } \mathrm{HH}_{i\pm1}) \\
PH_i^{t+1} &:= \mathrm{PTC}_i^t \text{ and } (\mathrm{HH}_{i-1}^t \text{ or } \mathrm{HH}_{i+1}^t) \\
SMO_i^{t+1} &:= \text{not } \mathrm{PTC}_i^t \text{ or } \mathrm{HH}_{i-1}^t \text{ or } \mathrm{HH}_{i+1}^t \\
ci_i^{t+1} &:= \text{not } \mathrm{EN}_i^t \\
CI_i^{t+1} &:= \mathrm{ci}_i^t \\
CIA_i^{t+1} &:= \mathrm{CI}_i^t \text{ and } (\mathrm{SMO}_i^t \text{ or } \mathrm{hh}_{i-1}^t \text{ or } \mathrm{hh}_{i+1}^t) \\
CIR_i^{t+1} &:= \mathrm{CI}_i^t \text{ and not } (\mathrm{SMO}_i^t \text{ or } \mathrm{hh}_{i-1}^t \text{ or } \mathrm{hh}_{i+1}^t)
\end{aligned}$$

Supplementary Table S4 - Wild Type and Knockout Initial and Attractor States

Wild type attractors

wg	0	1	0	0
WG	0	1	0	0
en	0	0	1	0
EN	0	0	1	0
hh	0	0	1	0
HH	0	0	1	0
ptc	0	1	0	1
PTC	1	1	0	1
PH	0	1	0	1
SMO	0	1	1	1
ci	1	1	0	1
CI	1	1	0	1
CIA	0	1	0	1
CIR	1	0	0	0
SLP	1	1	0	0

Wild Type Initial State

wg	0	1	0	0
WG	0	0	0	0
en	0	0	1	0
EN	0	0	0	0
hh	0	0	1	0
HH	0	0	0	0
ptc	1	1	0	1
PTC	0	0	0	0
PH	0	0	0	0
SMO	0	0	0	0
ci	1	1	0	1
CI	0	0	0	0
CIA	0	0	0	0
CIR	0	0	0	0
SLP	1	1	0	0

Knockout Experiment Attractor (en, hh, wg knockout)

wg	0	0	0	0
WG	0	0	0	0
en	0	0	0	0
EN	0	0	0	0
hh	0	0	0	0
HH	0	0	0	0
ptc	0	0	0	0
PTC	1	1	1	1
PH	0	0	0	0
SMO	0	0	0	0
ci	1	1	1	1
CI	1	1	1	1
CIA	0	0	0	0
CIR	1	1	1	1
SLP	1	1	0	0

Supplementary Figure S1 - Base model used in MC-Boomer search

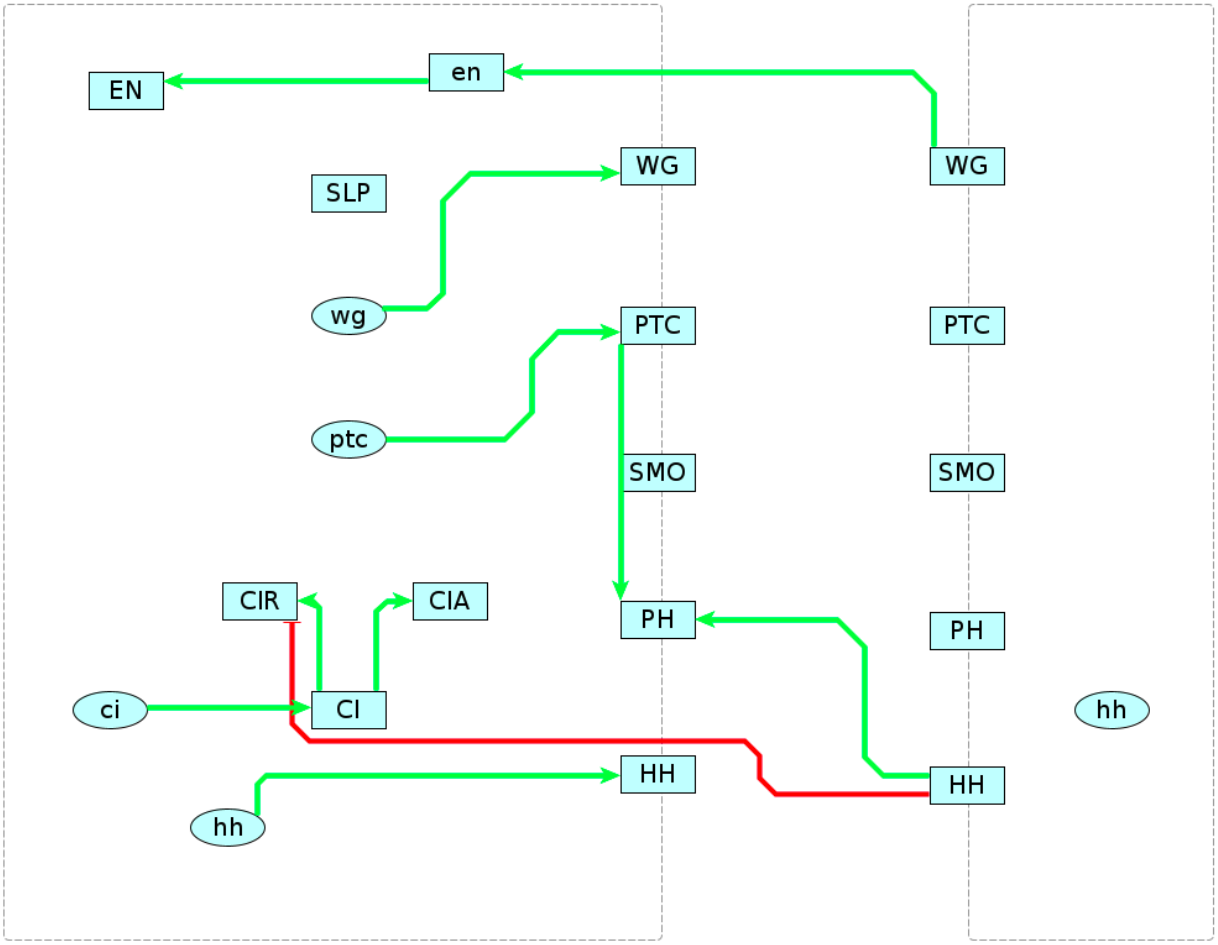
EN
en
SLP
WG
WG
wg
PTC
PTC
ptc
SMO
SMO
CIR
CIA
PH
PH
ci
CI
hh
HH
HH
hh
